# Production of O Radicals from Cavitation Bubbles under Ultrasound

**DOI:** 10.3390/molecules27154788

**Published:** 2022-07-26

**Authors:** Kyuichi Yasui

**Affiliations:** National Institute of Advanced Industrial Science and Technology (AIST), Nagoya 463-8560, Japan; k.yasui@aist.go.jp

**Keywords:** ultrasound, sonochemistry, O radicals, OH radicals, hydrogen peroxide, numerical simulations, ordinary differential equation (ODE), bubble, lifetime, ground state

## Abstract

In the present review, the production of O radicals (oxygen atoms) in acoustic cavitation is focused. According to numerical simulations of chemical reactions inside a bubble using an ODE model which has been validated through studies of single-bubble sonochemistry, not only OH radicals but also appreciable amounts of O radicals are generated inside a heated bubble at the violent collapse by thermal dissociation of water vapor and oxygen molecules. The main oxidant created inside an air bubble is O radicals when the bubble temperature is above about 6500 K for a gaseous bubble. However, the concentration and lifetime of O radicals in the liquid water around the cavitation bubbles are unknown at present. Whether O radicals play some role in sonochemical reactions in the liquid phase, which are usually thought to be dominated by OH radicals and $H_{2}O_{2}$, should be studied in the future.

## 1. Introduction

The reactivity of O radicals (oxygen atoms) in solution remains underexplored [1,2,3,4,5,6,7,8,9,10,11,12,13,14,15,16,17,18,19,20]. One of the reasons is the lack of a clean and mild method for the generation of O radicals in solution [1,2,3,4,9]. The generation of O radicals in solutions is commonly by UV irradiation of dibenzothiophene S-oxide (DBTO) [2,9,13]. The detection of O radicals in aqueous solutions may be possible by using the oxidation of thiol (RSH) as follows [2]:

RSH + O(^3^P) → RSOH (1)

RSOH + RSH → RSSR + H_2_O (2)

where O(^3^P) is the ground state O radical and RSSR is the observable disulfide product. It should be noted that non-volatile species should be used to probe O radicals in aqueous solutions under acoustic cavitation because volatile species enter cavitation bubbles and O radicals in the gas phase are detected instead of those in the liquid phase. In the present review, the possible production of O radicals in the liquid phase is discussed in relation to cavitation bubbles. With regard to cavitation bubbles, we will discuss them later in greater detail.

In typical advanced oxidation processes (AOP), the main oxidant is OH radicals [21,22,23,24,25]. The main oxidant in sonochemical reactions of liquid water irradiated by strong ultrasound has also been regarded as OH radicals [25,26,27,28,29,30,31,32,33]. When water is irradiated with strong ultrasound, many bubbles are created [34,35,36,37]. The bubbles expand during the rarefaction phase of ultrasound, and many of them violently collapse at the compression phase, which is called acoustic cavitation. The reason for the violent bubble collapse is as follows [34,35]: Due to the spherical geometry of the bubble collapse, the bubble wall speed freely increases as the bubble collapses because the surface area decreases and the inward liquid speed increases according to the continuity of the fluid (liquid). The other reason is the inertia of the inflowing liquid around the bubble. At the end of the violent bubble collapse, temperature and pressure inside a bubble increase to several thousand Kelvin and several hundreds of atmospheric pressure or more [34,35,38,39]. As a result, water vapor and oxygen (if preset) are dissociated inside the heated bubble and oxidants such as OH radicals, O radicals, H_2_O_2_ and O_3_ are formed inside the bubble [40,41]. The oxidants diffuse out of the bubble into the surrounding liquid and chemically react with solutes [27,28,34,40,42]. This is easily confirmed experimentally by the observation of the chemiluminescence of luminol (sonochemiluminescence) as well as the oxidation of I^−^ ions [33,43,44,45,46,47,48,49,50,51,52,53,54,55,56,57,58]. In the present review, the production of O radicals from cavitation bubbles under ultrasound is studied, particularly based on the results of numerical simulations of chemical reactions inside bubbles [40,41,59]. It is important because the main oxidant in sonochemical reactions may not always be OH radicals but sometimes O radicals, which may renew the interpretation of sonochemical reactions under some conditions.

## 2. Theoretical Model

**Figure 1:**
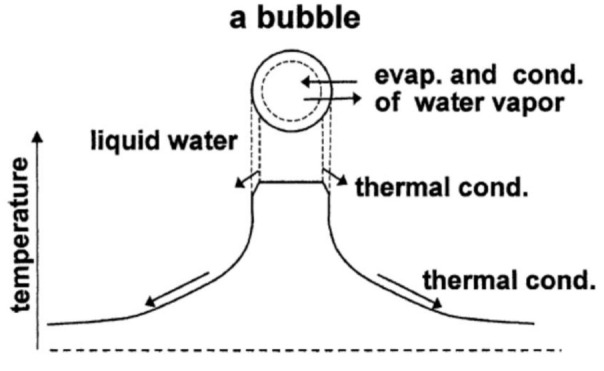
The model of bubble dynamics [67]. Copyright 2004, with permission from Elsevier.

The theoretical model of bubble dynamics has been developed through studies of single-bubble sonoluminescence (SBSL) and single-bubble sonochemistry [40,60,61,62]. SBSL is the light emission phenomenon from a single stably pulsating bubble trapped near the pressure antinode of a standing ultrasonic wave [63,64,65,66]. The light is emitted at each violent collapse of a bubble as a pulse due to the high temperature and pressure inside the bubble. In the theoretical model, pressure and temperature inside a bubble are assumed to be spatially uniform except at the thermal boundary layer near the bubble wall (Figure 1) [67]. In the model, the following effects are taken into account: non-equilibrium evaporation and condensation of water vapor at the bubble wall, thermal conduction both inside and outside a bubble, non-equilibrium chemical reactions inside a bubble, variation of liquid temperature at the bubble wall, the ionization of gases and vapor with ionization potential lowering caused by high density inside a bubble at the collapse, and the liquid compressibility to the first order of bubble wall speed divided by sound velocity in the liquid [40,60,61,62,68]. With regard to non-equilibrium chemical reactions inside a bubble, reaction rates of 93 chemical reactions and their backward reactions are numerically calculated involving N_2_, O_2_, H_2_O, OH, H, O, HO_2_, H_2_O_2_, O_3_, N, HNO_2_, HNO, HNO_3_, NO, NO_2_, and N_2_O for an air bubble [41]. Details of the chemical kinetics model are described in References [40,69,70,71,72]. Recently, however, Kalmár et al. [73] reported that the results of numerical simulations strongly depend on the chemical kinetics model used.

With regard to the assumption of the nearly spatially uniform temperature and pressure inside a bubble except at the thermal boundary layer, the full numerical simulations of the fundamental equations of fluid dynamics (partial differential equations (PDEs)) for gas inside a bubble have revealed that under many conditions temperature and pressure are spatially nearly uniform inside a bubble [74]. Thus, the present theoretical model consisting of ordinary differential equations (ODEs) is expected to work under many conditions. In general, ODE modelling is sometimes superior to PDE modelling in that it is computationally more economical and that the important factors are more easily traced [34]. Examples of other ODE modellings are described in References [75,76,77,78,79,80,81,82,83,84,85]. With regard to bubble dynamics models for chemical reactions inside a bubble, there are several other theoretical models [74,86,87,88,89,90,91,92].

## 3. Single-Bubble System

For the validation of the theoretical model, a single-bubble system is studied because direct comparison between experimental data and results of the numerical simulations is possible, as the acoustic amplitude at the position of a bubble is experimentally measured and the bubble-bubble interaction is absent [40,93,94,95,96,97]. The bubble-bubble interaction is the influence of neighboring bubbles on the pulsation of a bubble, which is complex because the distance between the bubbles as well as the number of bubbles temporally changes.

Inside an SBSL bubble, the bubble content is initially air and water vapor. At each violent bubble collapse, nitrogen and oxygen chemically react inside the heated bubble, and NO_X_ and HNO_X_ are formed. As NO_X_ and HNO_X_ are soluble in water, they gradually dissolve into liquid water. Finally, only argon and water vapor remain inside a SBSL bubble because 1% of air in molar fraction is argon, which is chemically inert. This argon rectification hypothesis has been validated both theoretically and experimentally [63,98,99].

**Figure 2:**
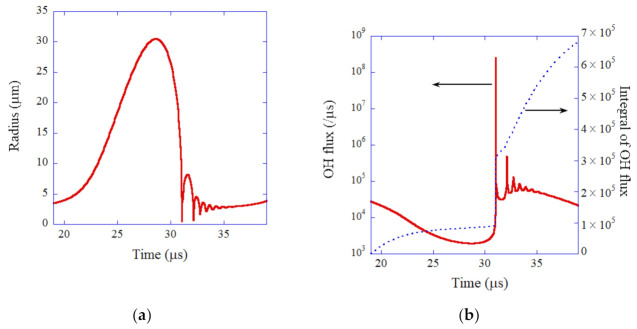
The results of the numerical simulation under the experimental condition of single-bubble sonochemistry as a function of time for one acoustic cycle [40]. The frequency and pressure amplitude of ultrasound are 52 kHz and 1.52 bar, respectively. (**a**) The bubble radius. (**b**) The dissolution rate of OH radicals into the liquid from the interior of the bubble (red solid line) and its time integral (blue dotted line). Copyright 2005, with permission of AIP Publishing.

In 2002, Didenko and Suslick [93] experimentally reported in *Nature* that the amount of OH radicals produced from the single-bubble system was 8.5 × 10^5^ molecules per acoustic cycle at the liquid temperature of 3 °C when the ultrasonic frequency and pressure amplitude were 52 kHz and 1.52 bar, respectively. The author [40] performed numerical simulations of chemical reactions inside an SBSL bubble in an experiment. A SBSL bubble, which is an argon bubble, expands during the rarefaction phase of ultrasound and collapses at the compression phase, followed by a small bouncing motion (Figure 2a) [40]. The calculated OH flux from the interior of a bubble to the surrounding liquid takes the maximum value at the end of the violent bubble collapse (Figure 2b) [40]. About one-third of the total amount of OH radicals which diffuses out of an SBSL bubble into the surrounding liquid in one acoustic cycle diffuses out of the bubble at the end of the violent bubble collapse. The other two-thirds diffuses out of the bubble during the bouncing motion and bubble expansion. The total amount of OH radicals which diffuses out of the bubble in one acoustic cycle (19.2 μs) is 6.6 × 10^5^ according to the numerical simulations [40]. It nearly agrees with the experimental data (8.5 × 10^5^). Thus, the theoretical model has been validated.

**Figure 3:**
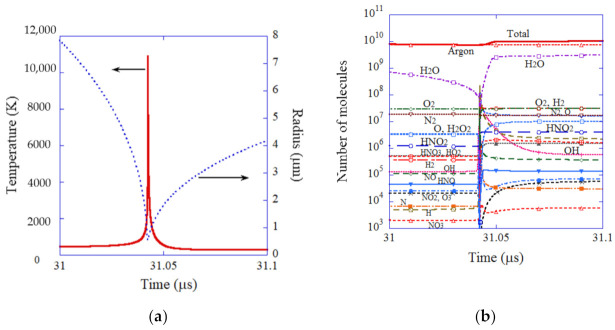
The results of the numerical simulations for an argon bubble under the condition of Figure 2 as a function of time at approximately the end of the violent bubble collapse for 0.1 μs [40]. (**a**) The bubble radius and the temperature inside a bubble. (**b**) The number of molecules inside a bubble. Copyright 2005, with permission of AIP Publishing.

Inside an SBSL bubble, temperature and pressure increase to 10,900 K and 7.9 × 10^9^ Pa, respectively at the end of the violent bubble collapse according to the numerical simulation (Figure 3a) [40]. The reason for the high bubble temperature is *pV* work by the surrounding liquid, which overwhelms the energy loss due to endothermic chemical reactions inside a bubble and thermal conduction from the heated interior of a bubble to the surrounding liquid [60]. As a result, many chemical species are generated inside an SBSL bubble by the dissociation of water vapor as well as that of nitrogen and oxygen which still remain inside an SBSL bubble in small amounts (Figure 3b) [40]. The main chemical products are H_2_, O, H_2_O_2_, H, HNO_2_, HO_2_, HNO_3_, and OH, in descending order according to the numerical simulation [40]. The amount of O radicals which diffuses out of an SBSL bubble into the surrounding liquid in one acoustic cycle is 1.3 × 10^7^ molecules, which is more than one order of magnitude larger than that of OH radicals [40]. The amount of the other oxidants is smaller than that of the O radicals H_2_O_2_: 6.3 × 10^6^ and O_3_: 3.4 × 10^4^ [40]. Thus, there is a possibility that O radicals are the main oxidant produced from an SBSL bubble rather than OH radicals. It should be noted that the amount of H_2_ (3.1 × 10^7^) is larger than that of O radicals in this case. Appreciable amounts of HO_2_ (1.1 × 10^6^) are also produced from an SBSL bubble. It means that superoxide radicals (O_2_^−^) could be formed in liquid water due to the following reaction: ${\mathrm{HO}}_{2}↔H^{+}+O_{2}^{-}$, where pK_a_(HO_2_) = 4.7 [41,100].

**Figure 4:**
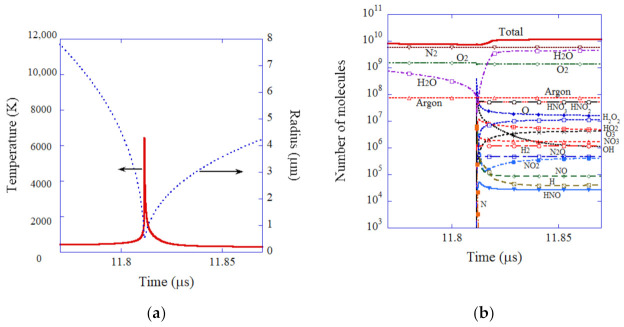
The results of the numerical simulation for an initial air bubble as a function of time at around the end of the violent bubble collapse for 0.1 μs [40]. (**a**) The bubble radius and the temperature inside a bubble. (**b**) The number of molecules inside a bubble. Copyright 2005, with permission of AIP Publishing.

For an initial air bubble, temperature inside a bubble increases to 6500 K at the end of the violent bubble collapse (Figure 4a) [40]. The bubble temperature in an air bubble is considerably lower than that in an argon bubble because the molar heat of air (mostly diatomic gases) is larger than that of argon (monatomic gas), as well as the fact that more endothermic chemical reactions occur inside an air bubble. The main chemical products from an air bubble are HNO_2_, HNO_3_, O, H_2_O_2_, O_3_, HO_2_, NO_3_, H_2_, and OH, in descending order (Figure 4b) [40]. The amount of O radicals which diffuses out of an air bubble into the surrounding liquid in one acoustic cycle is 1.6 × 10^7^, which is more than one order of magnitude larger than that of OH radicals (9.9 × 10^5^) [40]. The amount of the other oxidants is smaller than that of O radicals; H_2_O_2_: 5.1 × 10^6^ and O_3_: 2.7 × 10^6^ [40]. Thus, as in the case of an SBSL bubble, the main oxidant may be O radicals rather than OH radicals. It should be noted that the amount of O radicals is smaller than that of HNO_2_ (4.0 × 10^7^) and HNO_3_ (3.7 × 10^7^) in this case. Appreciable amounts of HO_2_(2.3 × 10^6^) are also produced from an air bubble.

## 4. Conditions for O Radical Production

**Figure 5:**
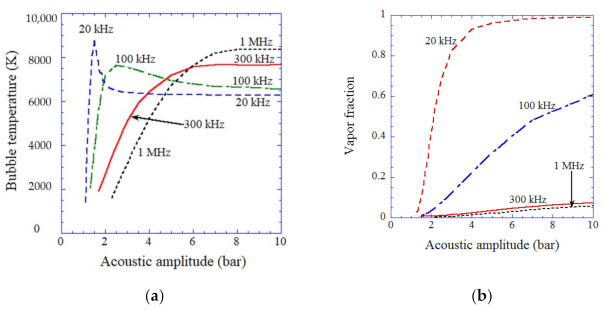
The results of the numerical simulations as a function of acoustic amplitude for various ultrasonic frequencies for the first collapse of an isolated air bubble [41]. The ambient bubble radii are 5 μm for 20 kHz, 3.5 μm for 100 and 300 kHz, and 1 μm for 1 MHz. (**a**) The temperature inside a bubble at the violent collapse. (**b**) The molar fraction of water vapor inside a bubble at the end of the violent collapse. Copyright 2007, with permission of AIP Publishing.

The main oxidant produced from acoustic cavitation bubbles depends on the conditions of ultrasonic frequency, acoustic pressure amplitude, ambient bubble radius, etc. according to the numerical simulations [41,59]. Firstly, the influence of ultrasonic frequency and acoustic pressure amplitude is discussed [41]. The temperature inside an air bubble at the violent bubble collapse is plotted as a function of acoustic pressure amplitude for various ultrasonic frequencies (20 kHz, 100 kHz, 300 kHz, and 1 MHz) according to the results of numerical simulations in Figure 5a [41]. For relatively low ultrasonic frequencies (20 kHz and 100 kHz), there is a peak in bubble temperature as a function of acoustic pressure amplitude; 8900 K at 1.5 bar for 20 kHz, and 7700 K at 2.5 bar for 100 kHz [41]. The reason for the existence of the peak bubble temperature is as follows. Initially, the bubble temperature increases as the acoustic pressure amplitude increases because a bubble expands more and the bubble collapse becomes more violent. As the acoustic pressure amplitude further increases, however, the molar fraction of vapor at the end of the bubble collapse increases because a bubble expands more and more amount of water vapor evaporates into a bubble during the bubble expansion. As the bubble collapse is very fast, condensation of water vapor at the bubble wall during the bubble collapse becomes strongly in non-equilibrium [68]. Thus, as the amount of water vapor evaporating into a bubble during the bubble expansion increases, the amount of water vapor trapped inside a bubble at the end of the bubble collapse increases. As the molar fraction of water vapor increases, the bubble temperature at the end of the bubble collapse decreases because of the endothermic dissociation of water vapor inside the heated bubble and the larger molar heat of water vapor (triatomic molecule) than that of air (diatomic molecule). Accordingly, there appears a peak in bubble temperature as a function of acoustic pressure amplitude for relatively low ultrasonic frequencies. For higher ultrasonic frequencies, on the other hand, bubble temperature continuously increases as the acoustic pressure amplitude increases, and finally reaches a plateau (Figure 5a). In this case, the bubble expansion is much less than that at lower ultrasonic frequencies because the acoustic period is much shorter. As a result, the amount of water vapor evaporating into a bubble during the bubble expansion is much less than that at lower ultrasonic frequencies. Accordingly, the molar fraction of water vapor at the end of the bubble collapse becomes very small (Figure 5b). Thus there is no peak in bubble temperature as a function of acoustic pressure amplitude for the higher ultrasonic frequencies.

When the molar fraction of water vapor inside a bubble at the end of the violent bubble collapse is more than 0.5, such a bubble is called a vaporous bubble [41]. When the vapor fraction is much less than 0.5 at the end of the violent collapse, such a bubble is called a gaseous bubble [41]. According to the results of numerical simulations shown in Figure 5b, a vaporous bubble appears for relatively low ultrasonic frequencies (20 kHz and 100 kHz) at relatively high acoustic amplitudes.

**Figure 6:**
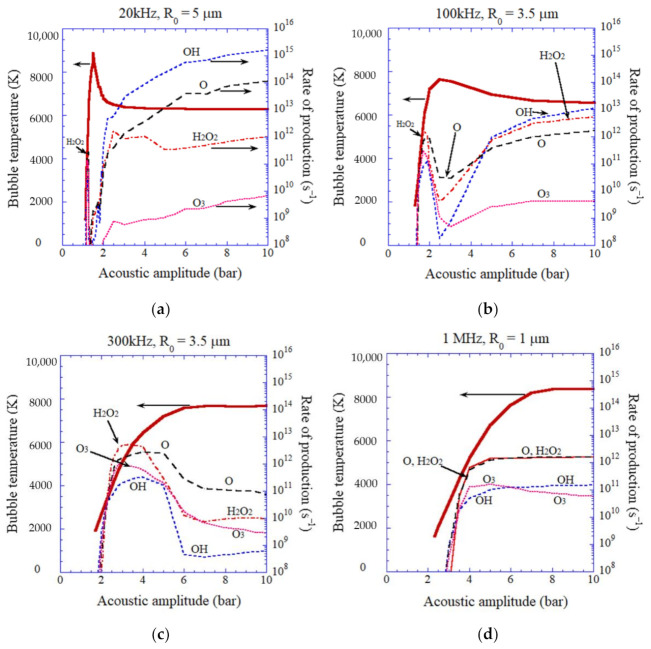
The results of the numerical simulations on the rate of production of each oxidant inside an isolated air bubble calculated by the first bubble collapse as a function of acoustic amplitude as well as the temperature inside a bubble at the end of the bubble collapse (the red thick line) [41]. (**a**) 20 kHz, (**b**) 100 kHz, (**c**) 300 kHz, (**d**) 1 MHz. Copyright 2007, with permission of AIP Publishing.

In Figure 6, rates of production of each oxidant inside an air bubble are plotted as a function of acoustic amplitude for various ultrasonic frequencies according to the numerical simulations [41]. As seen in Figure 4b, the number of molecules for each chemical species becomes nearly constant after about 0.05–0.1 μs after the end of the violent bubble collapse. The rates of production of each oxidant inside an air bubble shown in Figure 6 are calculated by the amount of each oxidant created inside an air bubble after about 0.05–0.1 μs after the end of the first violent bubble collapse multiplied by ultrasonic frequency.

From Figure 6a,b, the main oxidant created inside an air bubble is OH radicals for vaporous bubbles. This is because OH radicals are mostly created from water vapor [40]; $H_{2}O+M\to \mathrm{OH}+H+M$, $H_{2}O+O\to \mathrm{OH}+\mathrm{OH}$, $H_{2}O+H\to \mathrm{OH}+H_{2}$. From Figure 6a–d, the main oxidant is H_2_O_2_ for gaseous bubbles when the bubble temperature at the collapse is in the range of 4000–6500 K [41]. When the bubble temperature is above about 6500 K, the main oxidant is O radicals inside gaseous bubbles. O radicals are produced mainly by the following reactions; $O_{2}+H\to O+\mathrm{OH},{\;O}_{2}+M\to O+O+M,\;\mathrm{OH}+M\;\to O+H+M,$ and $H+\mathrm{OH}\;\to O+{\;H}_{2}$[41]. When the bubble temperature is higher than about 7000 K, the amount of all the oxidants become considerably smaller than that for moderately lower bubble temperatures because oxidants are strongly consumed inside an air bubble by oxidizing nitrogen [41,67]. Under the condition, the main chemical products inside an air bubble are HNO_2_, NO, and HNO_3_ [41].

**Figure 7:**
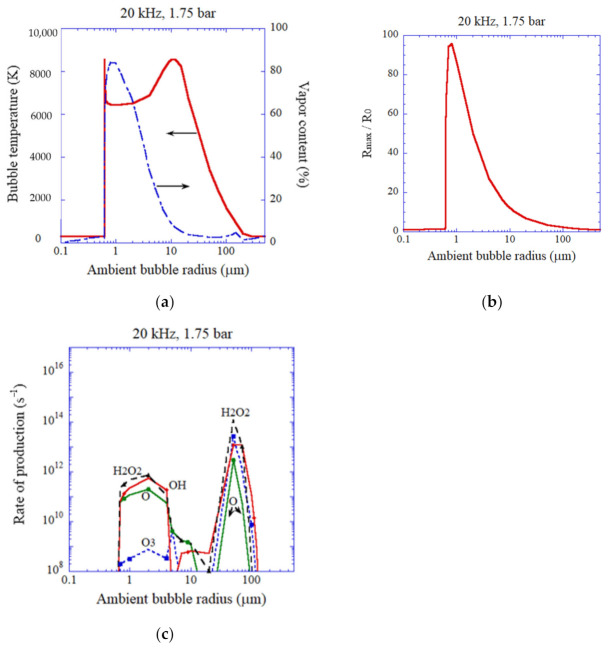
The results of the numerical simulations as a function of ambient bubble radius for an isolated air bubble when frequency and pressure amplitude of ultrasound are 20 kHz and 1.75 bar, respectively [59]. (**a**) The temperature inside a bubble and the molar fraction of water vapor at the violent collapse. (**b**) The expansion ratio. (**c**) The rate of production of each oxidant. Copyright 2008, with permission of AIP Publishing.

**Figure 8:**
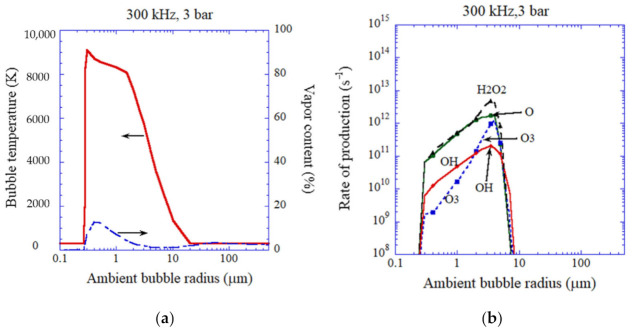
The results of the numerical simulations as a function of ambient bubble radius for an isolated air bubble for 300 kHz and 3 bar [59]. (**a**) The temperature inside a bubble and the molar fraction of water vapor at the violent collapse. (**b**) The rate of production of each oxidant. Copyright 2008, with permission of AIP Publishing.

In Figure 7 and Figure 8, the influence of ambient bubble radius, which is the bubble radius when ultrasound is absent, is shown according to the numerical simulations [59]. For 20 kHz and 1.75 bar in ultrasonic frequency and acoustic amplitude, respectively, shown in Figure 7, the bubble temperature at the violent collapse is nearly constant at 6500 K for the ambient bubble radius of 0.7 to 2 μm. It is because the molar fraction of water vapor inside a bubble is sufficiently high (vaporous bubble) due to the large expansion of a bubble during the rarefaction phase of ultrasound (Figure 7a,b). It is also seen in Figure 5a for 20 kHz at relatively high acoustic amplitudes. The detailed mechanism for the constant bubble temperature for the case of sufficiently high vapor fraction is discussed in Reference [101]. For the vaporous bubbles, the main oxidant is H_2_O_2_ and OH radicals in this case because the duration of high temperature inside a bubble is relatively short and there is not enough time for the dissociation of H_2_O_2_ inside a bubble; $H_{2}O_{2}+M\to 2\mathrm{OH}+M\;$ [41].

As the expansion ratio, which is defined by *R_MAX_/R_0_*, where *R_MAX_* is the maximum bubble radius and *R_0_* is the ambient bubble radius, decreases as the ambient bubble radius increases (Figure 7b), the vapor fraction at the end of the bubble collapse decreases (Figure 7a). As a result, the bubble temperature at the collapse increases to 8600 K at the ambient radius of 11 μm (Figure 7a) [59]. For the gaseous bubbles with higher temperature than about 7000 K, the amount of oxidant production becomes considerably smaller than that of the vaporous bubbles because oxidants are strongly consumed inside an air bubble by oxidizing nitrogen, as already discussed [41,67]. Under the condition, the main oxidants are O radicals, H_2_O_2_, and OH radicals (Figure 7c). For lower bubble temperature for gaseous bubbles, the main oxidants are H_2_O_2_ and OH radicals (Figure 7c).

For 300 kHz and 3 bar in ultrasonic frequency and acoustic amplitude, respectively (Figure 8), the vapor fraction is very small and bubbles are always gaseous. For relatively high bubble temperatures, the main oxidants are O radicals and H_2_O_2_ (Figure 8b). For relatively low bubble temperatures, the main oxidants are H_2_O_2_ and OH radicals. Appreciable amounts of O radicals and O_3_ are also created inside an air bubble under the condition. 

For an O_2_ bubble, on the other hand, the amount of O radicals produced inside a bubble is expected to be larger than that inside an air bubble because the consumption of O radicals inside an O_2_ bubble is less due to the absence of nitrogen [69]. 

According to numerical simulations by other groups [73,86,89,90,91,102,103,104,105,106,107,108], the production of an appreciable amount of O radicals inside a cavitation bubble has also been reported.

## 5. O Radicals in Liquid

**Figure 9:**
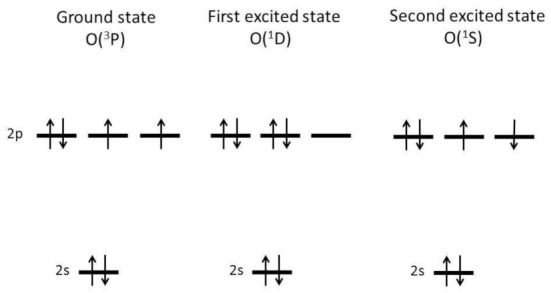
Electronic configurations of ground state, first excited state, and second excited state of the oxygen atom [109]. Two electrons in the 1s state are omitted. Copyright 2016, with permission from Springer.

An O radical (oxygen atom) has eight electrons. Two of them are in the 1s orbitals, and the other two electrons are in the 2s orbitals. The other four electrons are in the 2p orbitals if O radical is not highly excited (Figure 9) [109]. The orbitals are characterized by the principal quantum number (*n*), the orbital angular momentum (*l*), the magnetic quantum number (*m_l_*), and the secondary spin quantum number (*m_s_*) [110]. Orbitals are designated by s, p, and d corresponding to the orbital angular momentum *l* = 0,1,2, respectively. A 2p orbital means that *n* = 2 and *l* = 1. The magnetic quantum number (*m_l_*) can take the values of −*l*,(−*l* + 1),…,(*l* − 1). Thus *m_l_* can take the values of −1, 0, and 1 for the 2p orbital. For each *m_l_* state, two electrons can occupy; up-spin (*m_s_* = 1/2) and down-spin (*m_s_* = −1/2).

There are only three configurations (as shown in Figure 9) when four electrons occupy 2p orbitals [109,111,112]. The ground state is the configuration with the highest multiplicity according to Hund’s first rule. Thus, the configuration at the left side of Figure 9 corresponds to the ground state because the multiplicity is the highest due to the non-zero total spin angular momentum (*s* = |∑*m_s_*| = 1, and the multiplicity is 3). For the other two configurations in Figure 9, the total spin angular momentum is zero, and the multiplicity is 1.

The structure of four electrons in 2p orbitals is the same as that of two electrons in 2p orbitals because it is equivalent to the closed-shell structure (six electrons in 2p orbitals with *s* = *L* = 0, where L is the total orbital angular momentum) minus the structure of two electrons in 2p orbitals [112]. Accordingly, the total orbital angular momentum can take the values of *L* = 0, 1, and 2 because the allowed values of the total angular momentum for the system of two angular momenta of *j_1_* and *j_2_* are *j* = *j_1_* + *j_2_*, *j_1_* + *j_2_* – 1,…, |*j_1_* − *j_2_*| according to quantum mechanics [113]. The orbitals for *L* = 0, 1, 2, 3 are referred to as S, P, D, F, respectively [114]. For the configuration of the left side of Figure 9, *L* = 2 is impossible due to the Pauli’s exclusion principle because two spins are parallel in spite of the fact that the orbital angular momenta are also in parallel [115]. It is known that L = 0 is also impossible for this case due to Pauli’s exclusion principle [112]. Accordingly, the ground state of an O atom (O radical) is for *L* = 1 and designated by ^3^P where the total spin angular momentum s is coded in the form of 2s+1 in the left superscript.

The first excited state of O atom is ^1^D because with the same multiplicity of 1 (*s* = 0) the configuration with the highest total orbital angular momentum (*L*) has the lowest energy. For the first excited state, one of the 2p orbitals is empty (Figure 9). Thus, it more easily undergoes bond-forming addition reactions than the ground state O atom. For example, the following reaction with water molecules is known to be very fast [116,117].

(3) OD1+ H2O→H2O2

On the other hand, the ground state O(^3^P) is a selective oxidant because it relatively very slowly reacts with molecules that have no unpaired electrons such as ${\;H}_{2}O$ because such reactions violate the principle of spin conservation [117]. With molecules that have unpaired electrons, O(^3^P) rapidly reacts. For example, O(^3^P) immediately reacts with ground state oxygen molecules (triplet oxygen which has two unpaired electrons) as follows [117].

(4) OP3+ O2→ O3

**Figure 10:**
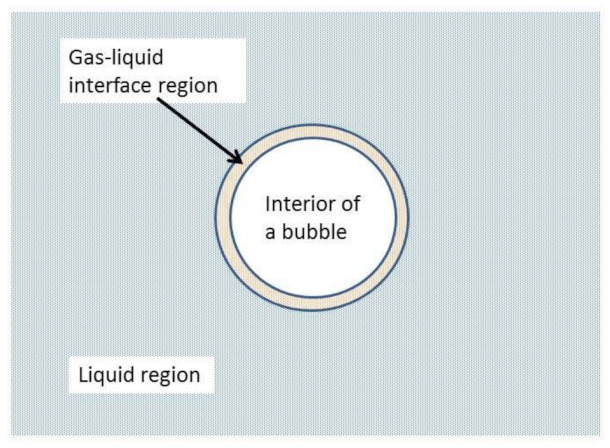
Three regions for a cavitation bubble [109]. Copyright 2016, with permission from Springer.

As discussed in the Introduction, however, the reactivity of O atoms (O radicals) in liquid water remains underexplored. With regard to the sonochemical reactions, it is unknown whether the produced O radicals from the interior of cavitation bubbles are mostly in the ground state O(^3^P) or in the first excited state O(^1^D). For O(^1^D) the reactivity with water is very high, and they can exist only at the gas-liquid interface region of a bubble (Figure 10) [109]. For O(^3^P), on the other hand, they may enter the liquid region as the reactivity with water is much lower (Figure 10). In this case, the lifetime of O radicals may be determined by some of the following reactions in the liquid phase in the absence of solutes; $O+O\;\to {\;O}_{2}$, $O+\mathrm{OH}\;\to H+{\;O}_{2}$, and O+H →OH. As the oxidation potential of O radicals is nearly comparable to that of OH radicals (Table 1) [118], it may be possible that O radicals play an important role in sonochemical reactions. With regard to OH radicals, the lifetime in liquid water near the bubble wall is about 20 ns, and they could be present in the gas-liquid interface region as the diffusion length is about 13 nm [34]. The lifetime of OH radicals is determined by the following reaction [34].

(5) $$\mathrm{OH}+\mathrm{OH}\;\to {\;H}_{2}O_{2}$$

The concentration of OH radicals near the bubble wall is experimentally determined as about 5 × 10^−3^ mol/L [27]. With regard to H_2_O_2_, the lifetime in liquid water could be as long as several days [119,120]. The lifetime of H_2_O_2_ in liquid water may be determined by the following decomposition [109,121].

(6) $$H_{2}O_{2}\;\to \;\frac{1}{2}{\;O}_{2}+{\;H}_{2}O$$

Accordingly, H_2_O_2_ could be present not only at the gas-liquid interface region but also in the liquid region. The typical concentration of H_2_O_2_ produced by acoustic cavitation bubbles is in the order of 10 μM (=10^−5^ mol/L) per hour [34,55]. With regard to O_3_, the life-time in liquid water is unclear at present [119].

**Figure 11:**
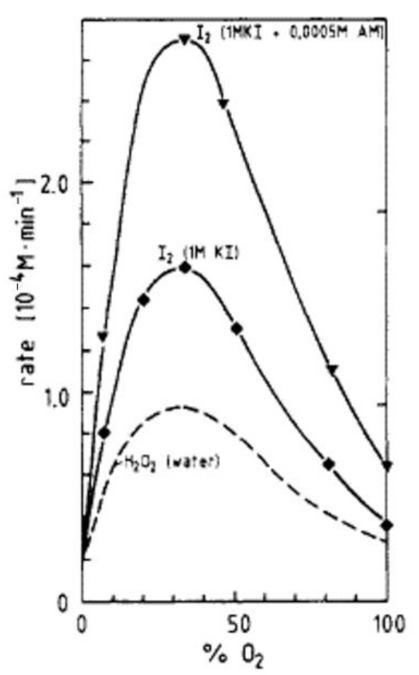
Experimental results on the rate of production of H_2_O_2_ in pure water and that of $I_{2}$ in 1 M KI solution or 1 M KI + 0.0005 M ammonium molybdate solution under various mixtures of argon and oxygen dissolved in the solution irradiated with ultrasound of 300 kHz [122]. Copyright 1985, with permission from the American Chemical Society.

Indeed, Hart and Henglein [122] experimentally suggested that O radicals play some role in sonochemical oxidation of KI (I^−^ ions) because the amount of KI oxidation was considerably larger than that of H_2_O_2_ formation in pure water (Figure 11). KI oxidation is usually considered as a result of the following reaction with OH radicals.

(7) $$2\mathrm{OH}+2I^{-}\to {\;I}_{2}+2{\mathrm{OH}}^{-}$$

In the presence of ammonium molybdate as a catalyst, the following reaction occurs.

(8) $$H_{2}O_{2}+2I^{-}\to {\;I}_{2}+2{\mathrm{OH}}^{-}$$

The discrepancy between the amount of KI oxidation and that of H_2_O_2_ formation in pure water is possibly due to the following reaction of O radicals [109,122].

(9) $$O+2I^{-}+2H^{+}\;\to {\;I}_{2}+{\;H}_{2}O$$

However, there has been no direct experimental evidence for the production of O radicals in the liquid phase from cavitation bubbles. The role of O radicals in chemical reactions in the liquid phase also remains unexplored in the field of plasma-liquid interactions [123].

## 6. Conclusions

Numerical simulations of chemical reactions inside a cavitation bubble under ultrasound have indicated that appreciable amounts of O radicals are produced from cavitation bubbles. In particular, O radicals may be the main oxidant produced from gaseous bubbles when the bubble temperature at the violent collapse is higher than about 6500 K. Although the first excited state O atom (O(^1^D)) could be only present at the gas-liquid interface region of a bubble due to the extreme high reactivity with water, the ground state O atom (O(^3^P)) may enter the liquid region as the reactivity with water is much lower. There has been no direct experimental evidence of O radical production in the liquid phase by cavitation bubbles so far. Further studies on this topic are required.

## Figures and Tables

**Table 1:** Oxidation potential of oxidants [118].

Oxidant	Reaction	Oxidation Potential (V)
OH	$\mathrm{OH}+{\;H}^{+}+e\;\to {\;H}_{2}O$	2.81
O	$O+2H^{+}+2e\;\to {\;H}_{2}O$	2.42
O_3_	$O_{3}+2H^{+}+2e\;\to {\;O}_{2}+{\;H}_{2}O$	2.07
H_2_O_2_	$\frac{1}{2}H_{2}O_{2}+{\;H}^{+}+e\;\to {\;H}_{2}O$	1.78

## Data Availability

Not applicable.
